# What is creating the height premium? New evidence from a Mendelian randomization analysis in China

**DOI:** 10.1371/journal.pone.0230555

**Published:** 2020-04-10

**Authors:** Jun Wang, Qihui Chen, Gang Chen, Yingxiang Li, Guoshu Kong, Chen Zhu

**Affiliations:** 1 School of Public Administration and Policy, Renmin University of China, Beijing, China; 2 Center for Health Policy Research and Evaluation, Renmin University of China, Beijing, China; 3 College of Economics and Management, China Agricultural University, Beijing, China; 4 WeGene, Shenzhen, China; University of Jyvaskyla, FINLAND

## Abstract

This study uses a Mendelian randomization approach to resolve the difficulties of identifying the causal relationship between height and earnings by using a unique sample of 3,427 respondents from mainland China with sociodemographic information linked to individual genotyping data. Exploiting genetic variations to create instrumental variables for observed height, we find that while OLS regressions yield that an additional centimeter in height is associated with a 10–13% increase in one’s annual earnings, IV estimates reveal only an insubstantial causal effect of height. Further analyses suggest that the observed height premium is likely to pick up the impacts of several cognitive/noncognitive skills on earnings confounded in previous studies, such as mental health, risk preference, and personality factors. Our study is the first empirical study that employs genetic IVs in developing countries, and our results contribute to the recent debate on the mechanism of height premium.

## 1. Introduction

Height is widely believed to be a key determinant of professional success. Indeed, taller people are usually found to earn more in both developed countries [1–5] and developing countries [6–10]. Yet why is a height-wage premium observed? More intriguingly, why is it observed even in labor markets where height is unlikely to be a crucial productive factor, say, sedentary white-collar jobs in industrialized countries? This question has led many researchers to interpret the observed height premium as evidence of labor-market discrimination by employers who stigmatize shorter people [11–12]. However, this interpretation has recently been challenged on the ground that the observed link between height and better labor-market performance may reflect the influence of unobserved factors (that affect one’s labor-market performance). For example, height has been found to be correlated with both cognitive skills [1–2,13–14] and non-cognitive skills [14–15], skills that contribute greatly to one’s labor-market success [16].

These differing interpretations point into vastly different directions of policy-making. If labor-market discrimination is at work, then policies aiming to prevent discrimination may be needed to protect employee welfare. In contrast, if the observed height premium is, in fact, capturing the impact of some hard-to-observe factors, such as emotional stability and perseverance, then using height as a marker to gauge returns to these factors is justifiable (and may even be desirable). Therefore, to better inform policy in response to inequality, it is crucial to investigate the origins and driving channels of the observed height premium. Such an investigation is of particular interest in transitory economies, where the role of height as a productive factor is rapidly changing, as a result of structural transformations undergoing in the labor market (e.g., changes in the relative shares of blue-collar and white-collar jobs) and in the economy as a whole (e.g., changes in the relative shares of different sectors).

China provides an interesting case to study. Partly due to China’s market-oriented reform, employment opportunities and wage determination rely more on the market rather than institutional and political factors [17–18], which leads to complex implications for the role height plays in wage determination. On the one hand, as the Chinese economy advances and its industrial structure upgrades, demand for unskilled labor has been falling since the early 1980s [19], which downplays the earning potential of physical strength. On the other hand, the rapid development of the service sector in the recent decade, exemplified by the booming express delivery services for on-line purchased daily and food products, creates new room for physical strength and endurance to play a role. Echoing such complexity, empirical studies conducted in China usually find that while the observed height premium does pick up some impacts of factors such as political capital, it does not entirely vanish after these factors have been controlled for in estimation [20–21]. Thus, it is natural to ask: to what extent is height itself rewarded in China’s labor market? And to what extent does the height premium capture the impact of other factors?

Needless to say, estimating the causal effect of height on wages using observational data is challenging, as it is difficult to control for all potential confounders in estimation. A standard solution is to find instrumental variables (IVs) for height, i.e., exogenous variables that affect the wage only through their impacts on height, to achieve identification. Some researchers exploited twinning experiments to identify the impact of height [22]. Since identical twins share the same genes and similar family environment, it is possible to eliminate shared environmental factors, such as the family background, neighborhood and peer effects, and genetic factors, using twin data. However, the external validity of twin studies is usually quite limited, since twins account for only 2% in any human population. Previous studies conducted in developing countries routinely used shocks to childhood nutrition, such as occurrences of droughts or disease outbreaks, relatives’ height or community-level average height, to identify the impact of height. Yet the effect so identified mainly captures the impact of (the part of height that is determined by) childhood environmental factors, which may be correlated with other unobserved productive factors whose impacts we would like to purge out in estimation.

Taking another path, this paper adopts an IV strategy based on genetic information to achieve identification. Similar to those recently adopted by von Hinke et al. [23], Tyrrell et al. [24], and Böckerman et al. [25], we exploit variations in individuals’ genetic markers to create an IV for height within the framework of Mendelian randomization (MR) [26]. To the extent that genetic markers strongly predict one’s adult height but do not directly affect one’s labor-market performance, they provide a source of exogenous variations in height needed for identification. More specifically, we instrument height using the polygenic score with gene variations that have been found to be significantly associated with height in genome-wide association studies (GWAS) using extensive population samples [27]. Polygenic scores (PGSs; also called "polygenic risk scores," "genetic risk scores," or "genome-wide scores") are aggregated effects of hundreds and thousands of trait-associated DNA variants identified in GWAS studies and can be used to predict propensities toward certain traits and outcomes [28–29]. While GWAS were mostly based on samples of European ancestry, recent studies indicate that the results can well apply to East Asian (e.g., Chinese) populations. For example, Duncan et al. [30] demonstrate that the polygenic score performance is reasonably reliable in East Asian samples (95%) relative to European samples (100%). However, the predictive performance of PGSs is much lower in African (42%) and South Asian (60%) samples.

By analyzing a unique dataset recently collected in China, we find that the statistically and economically significant height premium estimated by OLS regressions, i.e., an additional centimeter in height is associated with a 10–13% increase in one’s annual earnings, largely reflects impacts of other factors. While our OLS estimates are similar to their counterparts in most previous studies conducted in China [20–21], our IV estimates reveal only a negligible causal impact of height. Further analysis reveals that the observed height premium is likely picking up the impacts of several cognitive/non-cognitive skills on earnings (e.g., mental health, risk preference).

Our analysis makes three contributions to the literature. First, our study is the first height-premium study that employs genetic IVs in China, if not the first in developing countries. Not only can our findings provide a deeper understanding of the working of China’s labor market, but they are also complementary to findings in other, more developed countries [23–25]. Second, our findings are complementary to the recent discovery that the observed height premium largely captures the impact of other factors, rather than a reflection of labor-market discrimination believed by many previous researchers. Third, we extensively test the relevance, independence, and exclusion restrictions of the genetic instrument of height. The demonstration of the instrument validity not only strengthens our own findings, but also lends support to previous findings that are based on such genetic IV.

The remainder of this paper is structured as follows. The next section briefly reviews the relevant literature, paying particular attention to the channels through which height may affect one’s wage income. Section 3 describes the data. Section 4 details our empirical methods. Section 5 presents our main empirical results. The final section draws conclusions and points out a number of directions for future research.

## 2. Relevant literature

### 2.1. Why may height affect wages?

To better understand what genetic IVs can identify, it is helpful to consider first the potential channels through which height may affect wages. Thus far, four channels have been put forward in the literature. First of all, height itself may be a productive factor, especially in developing countries where many jobs value physical strength and endurance of physical labor [31–33]. Yet these physical features are less critical for sedentary, white-collar jobs in more developed settings.

Secondly, height may be the *generator* of some other productive factors. For example, taller people may enjoy more social dominance [34–35] and have higher self-esteem [36], all of which may improve their competitiveness and labor-market performance [37]. The height premium may also be generated through *perception* and *expectation*. For example, taller people may be *perceived* as more productive by employers [38]. The perceived productivity of taller people may, in turn, influence the expectation of their employers, inducing them to assign more challenging and high-return tasks to taller employees [39]. Thus taller employees may have more opportunities to outperform their shorter counterparts in the labor market through a self-fulfilling prophecy effect. To the extent that these channels also reflect the productive effects of height, their existence should not be considered as evidence of labor-market discrimination.

Thirdly, height may be correlated with productive factors that are not generated by itself. The most well-known examples include cognitive skills [1] and non-cognitive skills [15]. While taller children tend to develop more cognitive [21,40] and non-cognitive skills [14], these skills need not be products of height *per se* to act as confounders in a conventional height-wage regression. As illustrated in Case and Paxson [1], for example, both height and these skills could be outcomes of some underlying *endowment*, say, insulin-like growth factors that stimulate simultaneous neural and physical growth [41–42], which is sufficient to generate a significant correlation between height and cognitive/non-cognitive skills in adulthood. Childhood environmental factors, such as food abundance in childhood, parental education, and household income, can also lead to correlations between height and cognitive developments [14]. Height may even be correlated with other types of human capital, such as political capital. Yamamura et al. [21], for example, show that taller youth in China are more likely to join the Chinese Communist Party (CCP) and CCP membership is rewarded in the labor market.

Finally, the observed height premium may simply reflect pure discrimination against shorter employees in the labor market [11–12,43]. Under pure discrimination, taller employees are paid more for reasons that are not related to their productivity, whether actual or perceived.

### 2.2. Recent methodological development

**Fig 1:**
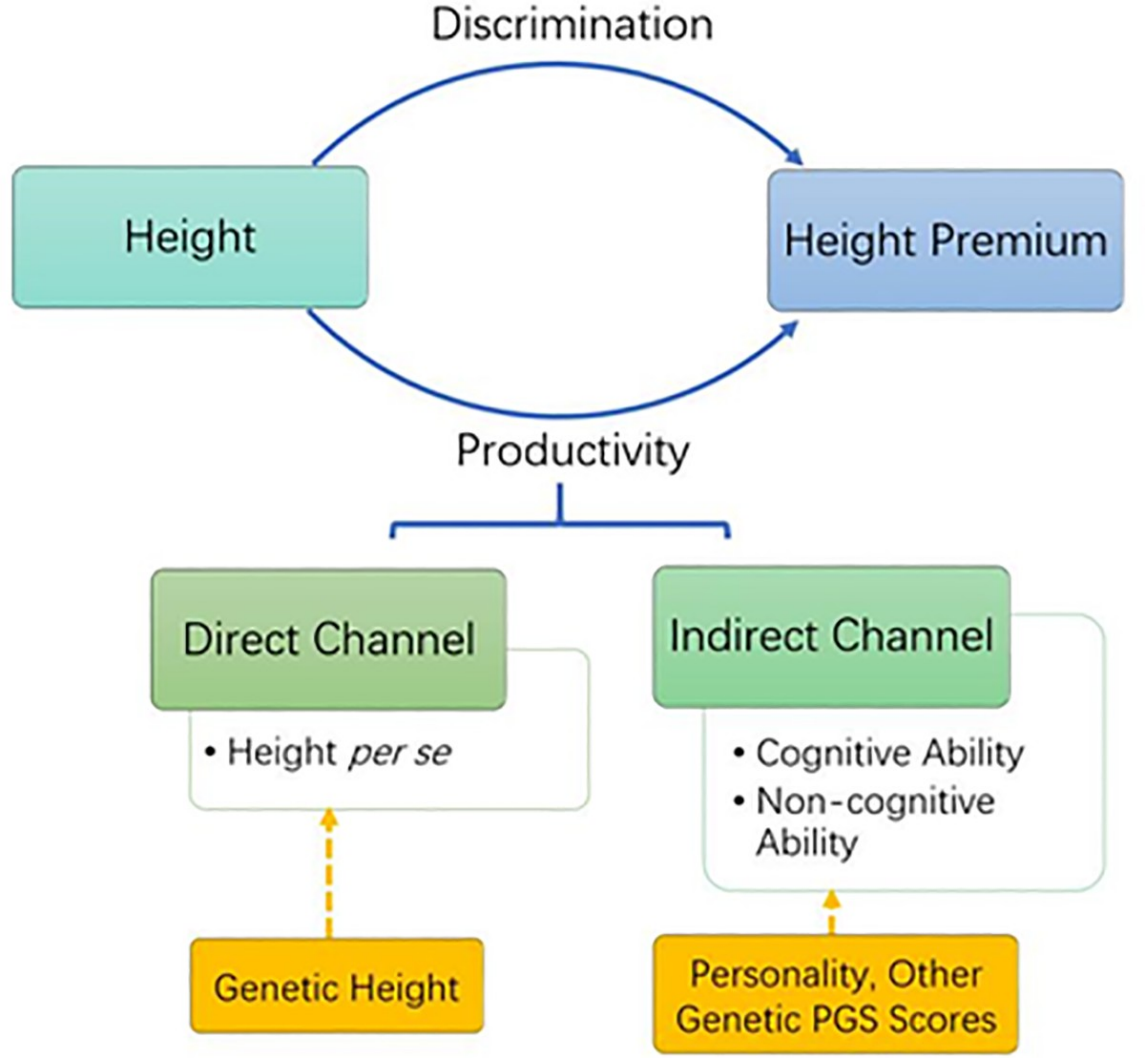
Channels identified using genetic instruments.

While earlier studies tend to examine what the height premium captures in an indirect manner, estimating OLS models that control for variables capturing impacts through the afore-mentioned channels, more-recent studies tend to estimate the impact of genetic height on wages more directly. The recent decade witnessed a new strand of studies that employ variations in one’s genetic markups induced by Mendelian randomization to identify the impact of height [23–25], which is the impact of height working through the first two channels discussed above (Fig 1). More specifically, these studies instrument height using the polygenic score with gene variations that have been found to be significantly associated with height in genome-wide association studies based on extensive population samples [27]. An empirical regularity emerging from these studies is that the impacts of height identified by genetic variations are routinely much smaller than their OLS counterparts.

The present paper also exploits genetic variations to identify the causal impact of height in the context of China for the first time. We control for several personality characteristics as well as additional genetic factors, which allows us to see more clearly the role of height in wage determination.

## 3. Data

### 3.1. Database and survey

Our empirical analysis is based on a unique dataset collected in collaboration with WeGene, a leading private genetic testing company founded in 2014 in Shenzhen, China. The study has been approved by the Institutional Review Board of College of Economics and Management at China Agricultural University (CAU-CEM-IRB; approval number: HC0028401A). All participants of this study were drawn from the customer database of WeGene, all of whom provided (signed) informed consent and participated in the online genoeconomic survey sometime between April 2018 and January 2019. Approximately 3,600 participants completed the full survey. The online survey collected information on each participant’s demographic characteristics such as height, year and month of birth, their socioeconomic characteristics such as annual income and educational attainment, as well as their preferences such as risk attitude, altruism and trust [44–45].

Important to our study, all sample subjects were genotyped on a WeGene custom genotyping array platform (AffyMetrix). Imputation and quality control were performed by WeGene using PLINK (1.90 Beta), SHAPEIT (v2.17) and IMPUTE2 (v2.3.1). A total of 10,670,107 Single Nucleotide Polymorphisms (SNPs) were identified for each participant, which were then used to construct polygenic scores and genetic instrumental variables. Respondents’ economic preferences (risk-loving, altruism and trust) were assessed using the Global Preferences Survey on a 10-point scale [45].

### 3.2. Sample characteristics

**Fig 2:**
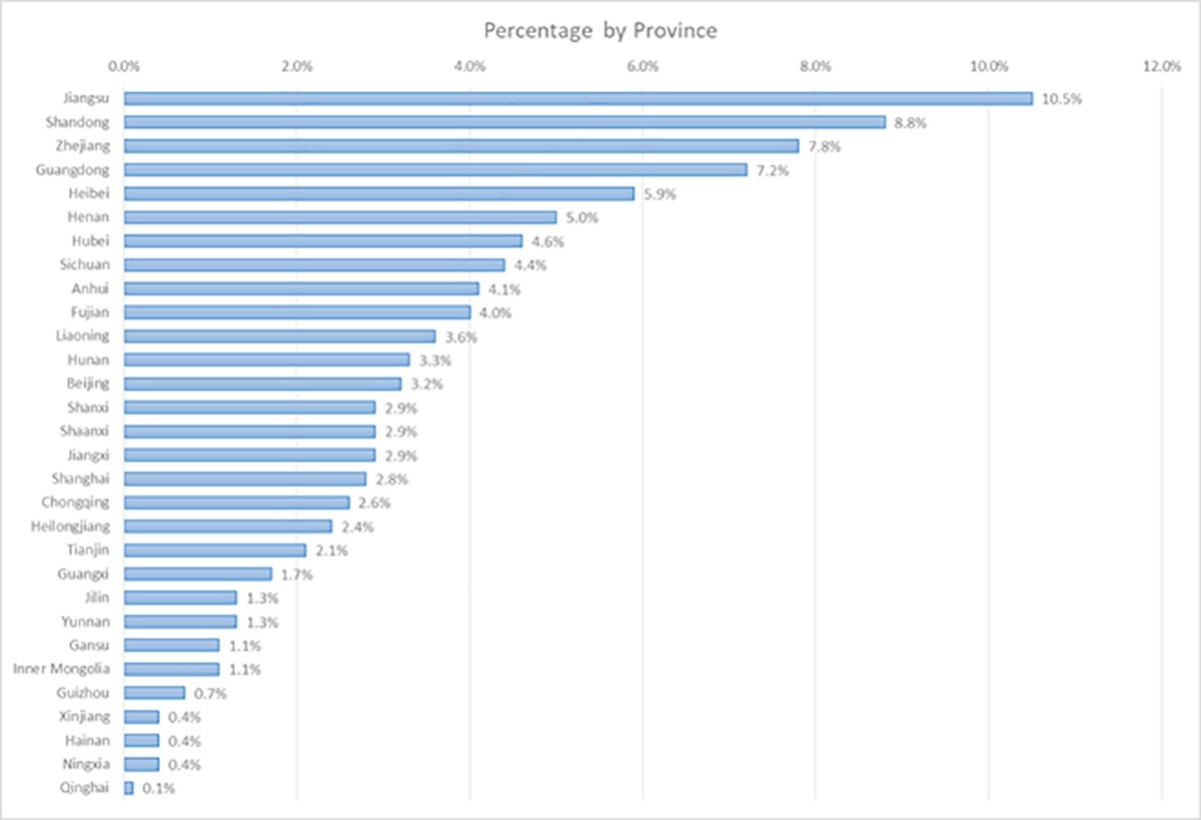
Spatial distributions of observations.

Excluding individuals who were younger than the age of 16, older than 60, or still a student at the time of the survey from the original dataset yields an analytical sample with 3,427 observations (1,922 males and 1,505 females). Table 1 provides summary statistics of the major characteristics of respondents in the analytical sample. Consistent with previous findings that Direct-to-Consumer genetic testing customers are generally well-educated middle-class professionals [46], an average respondent in the sample earned CNY138,927 (1 US Dollar ≈ CNY 6.8) in 2017 and completed 15.6 years of education, both being significantly higher than the state-wide averages. The average annual wage of an urban employee is CNY 74,318 in 2017 (Source: http://www.chinadaily.com.cn/a/201805/21/WS5b02d4d6a3103f6866ee9b15.html). The average educational attainment of an employee is 10.2 years in 2015 (Source: http://www.gov.cn/xinwen/2017-07/25/content_5213292.htm). The spatial distribution of respondents’ home provinces, shown in Fig 2, also mirrors the above pattern: the top three provinces are Jiangsu, Shandong and Zhejiang, which are all located the most developed eastern coastal areas, whereas provinces in the less-developed western China (i.e., Ningxia and Qinghai) have the least respondents.

**Table 1:** Summary statistics of the analytical sample (N = 3,427).

Variable	Mean/Percentage	Std.Dev.
*Socio-demographic Characteristics*:		
Annual Earnings (in CNY)	138,927	179,554
Natural Logarithm of Annual Earnings	11.570	0.893
Age	30.245	6.667
Years of Schooling	15.604	2.237
Self-reported Height (in centimeter)	169.088	8.331
Male	56.1%	-
*Genetic Instrumental Variable*:		
Polygenic Score of Height [47]	-0.598	0.446
*Economic Preferences*:		
Risk Loving	5.613	2.136
Altruism	4.862	2.490
Trust	5.084	2.719
*Other Polygenic Score Controls*:		
Polygenic Score of Cognitive Ability [48]	-0.010	0.171
Polygenic Score of Depression [49]	32.628	4.233
Polygenic Score of Delay Discounting [50]	0.074	0.087
Polygenic Score of Reproduction Preference [51]	0.029	0.166

Source: Data are drawn from the consumer information base of WeGene. Summary statistics are calculated by the author.

## 4. Empirical methods

Our empirical analysis mainly consists of two related parts. The first part measures the potential height premium in China’s urban labor market. The second part then seeks to explain what the observed height premium really captures by (a) sequentially adding a set of covariates in the model, and (b) instrumenting height using the genetic IV.

As a starting point, consider a statistical model that links one’s wage income and adult height:

$$\log\left(Y\right)=\beta_{0}+\beta_{1}H_{}+X\beta_{2}+u,$$ (1)

where *Y* is one’s annual earnings (in log), *H* is his or her adult height measured in centimeters (cm), **X** is a set of personal characteristics to be detailed below, and *u* is an error term capturing the influence of all unobserved factors. If Eq (1) is correctly specified, the coefficient on height, β_1_, captures the causal effect of being one cm taller on one’s earnings, which can be estimated by ordinary least-squares (OLS) techniques. However, as discussed above, in many situations, OLS estimates of β_1_ may capture something other than the causal effect of height.

### 4.1. Measuring the height premium

Assuming away identification problems with OLS for the moment, one subtle issue in estimating the height premium is: What covariates should be included in Eq (1)? A number of studies conducted in China adopted an extended version of Mincer’s earnings equation to capture the height premium [52], which includes years of schooling, post-school experience, experience squared, marital status, self-reported health, household registration (*Hukou*) status, political affiliation, as well as dummies for occupation, industry, and firm ownership [20–21], as covariates. However, as argued by Neal and Johnson [53], Heckman [54], and Persico et al. [15], many of these covariates, such as years of schooling and occupation, are *choice* variables that can be influenced by height or are jointly determined with height, and thus the inclusion of these variables may yield misleading results. Therefore, we adopt a specification similar to that used by Persico et al. [15] as the benchmark specification, which includes only gender and age as covariates. But for comparison and exploratory purposes, we expand the set of covariates sequentially to include proxies for skills such as years of schooling, measures of personal preferences such as risk preference, altruism and trust, as well as other genetic controls–i.e., polygenic scores of cognitive ability [48], depression [49], delay discounting [50], and reproduction preference [51]. Expanding the set of covariates sequentially provides an opportunity for us to see what is being captured by the height premium estimated in the benchmark model.

### 4.2. Genetic instrument: The polygenic score of height

Another issue is raised by the existence of unobserved confounders that are correlated with both one’s height and labor market performance. A standard solution is to find IVs for height, i.e., variables that are highly correlated with height (conditional on the covariates **X**) but are orthogonal to potential confounders, to eliminate the influence of confounders in estimating Equ (1). We construct an IV exploiting one’s genetic information.

The genetic IV is constructed based on the notion of “Mendelian randomization”, which refers to the random assignment of an individual’s genotype at conception [55–56]. Since adult height is known as one of the highest heritable traits in human beings, determined approximately 80% by genetic factors and 20% by environmental factors [57–58], genetic variations related to height can naturally serve as unconfounded proxies for observed adult height [23–26].

It is worth noting that there is no *single* gene for height in humans. Instead, there are hundreds of genes and DNA segments that are determining the adult height in human genome. In other words, the genetic basis of human height is quantitative, rather than qualitative. More specifically, this IV is constructed as the weighted polygenic score of height (*PGS_Height*) based on 697 SNPs previously identified to be associated with adult height in human beings [47]. Mathematically, *PGS_Height*_*i*_ is calculated as the sum of genotyped or imputed allele *j* dosages carried by a respondent *i* (*SNP*_*j*,*i*_) multiplied by the estimated effect size (*β*_*j*_) reported by Wood et al. [47] (We use the Affymetrix WeGene V1 Arrays to assay common SNP variation in the genome of all participants, and then imputed additional SNPs):

$${PGScore\_Height}_{i}=\sum_{j=1}^{J=697}\beta_{j}{SNP}_{j,i}$$ (2)

**Fig 3:**
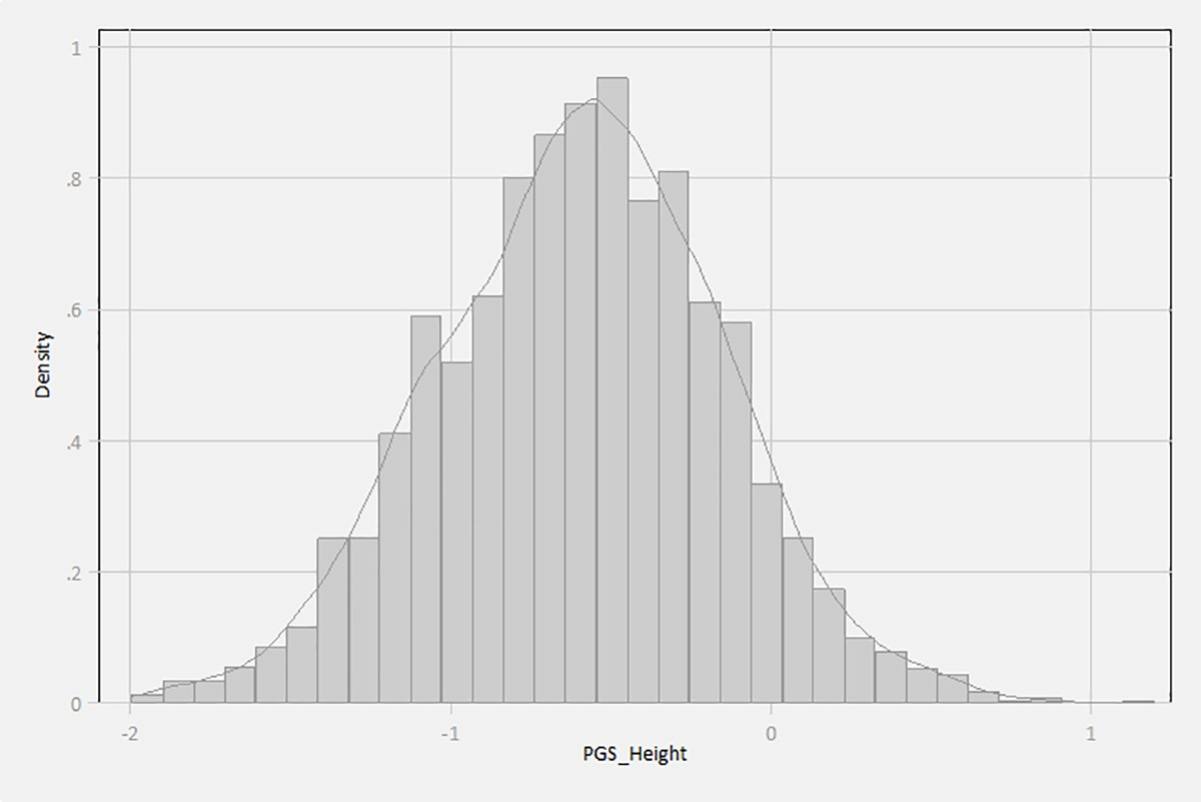
Distribution of the genetic instrumental variable.

Note that because each person can have 0, 1, or 2 risky (effect) allele(s) for each of the 697 SNPs, *SNP_j,i_* can be 0, 1, or 2. Fig 3 presents a plot of the distribution (with kernel-smoothed density) of the constructed polygenic score of height in our sample. As demonstrated in the plot, the genetic spectrum of *PGS_Height* could range from low to high, and the higher score that an individual has, the more likely that he/she would be taller.

Note that whereas Mendelian randomization is orthogonal to confounders that are correlated with but are not determined by height, it may not be orthogonal to those that are (at least partly) determined or triggered by height, such as social dominance and self-esteem discussed in Section 2.1. In this sense, the ‘causal’ effect of height identified using *PGS_Height* captures not only the impact of genetic height, but also the impact of social reactions that are *triggered* by genetic height–in other words, it serves as an upper bound of the economic returns to genetic height.

With the genetic IV discussed above, we estimate the following first-stage equation,

$$H=\alpha_{0}+G\alpha_{1}+X\alpha_{2}+v,$$ (3)

where **G** = *PGS_Height*_*i*_ is the genetic IV, along with Eq (1) in a two-stage least-squares (2SLS) framework.

Note finally that the above framework (Eqs 1–3) implicitly invokes the common-return assumption that the economic return to height is the same for all individuals in the underlying population–in other words, β_1_ does not have a subscript for individual *i*. von Hinke et al. [14,23] formally extended the genetic-IV framework to allow for heterogeneous return to height in the spirit of Imbens and Angrist [59] and Angrist et al. [60] and provided assumptions needed to identify these effects. Under the heterogenous-return framework, β_1_ captures the local-average treatment-effect (LATE) for those individuals who are taller *because* they have the particular genes that made them tall. In contrast, the impact of height identified in many previous studies in developing countries using shocks to childhood nutrition (e.g., occurrences of droughts or disease outbreaks) captures the LATE for those individuals who are shorter *because* they encountered these shocks.

### 4.3. Instrument validity

The validity and credibility of *PGS_Height* rely on several critical criteria that we discuss in detail below [25,61–63].

#### 4.3.1. Relevance

The relevance criterion requires that the genetic IV of PGS_Height must be correlated with the endogenous variable of observed height. In our data, the explanatory power of PGS_Height is 0.0459, comparable to the 0.048 that Böckerman and colleagues found using a Finnish sample [25]. We further test and confirm the association between PGS_height and observed height in the first stage regressions of 2SLS (reported in Table 7).

#### 4.3.2. Independence

The independence criterion requires that the genetic IV of PGS_Height must not be correlated with unmeasured confounders through population stratification. We first divide our analytical sample into two groups: the higher PGS_Height subsample with an above-average value and the lower PGS_Height subsample with a below-average value of the polygenic score. We then assess whether the two groups differ in major observable characteristics significantly. As reported in Table 2, the subsample averages are in general similar between the two groups, except for the endogenous variable of observed height, suggesting little correlation between the genetic IV and other observed variables.

**Table 2:** Comparison of observable characteristics by *PGS_Height* (n = 3,427).

Observable characteristics	Higher *PGS_Height*	Lower *PGS_Height*	Difference	t-statistics	p-value
Natural Logarithm of Annual Earnings	11.585	11.553	0.032	0.835	0.404
	(0.026)	(0.029)			
Height (in centimeter)	171.342	168.663	2.679	8.109***	0.000
	(0.225)	(0.242)			
Age	30.150	30.353	-0.202	-0.756	0.450
	(0.186)	(0.191)			
Years of Schooling	16.429	16.445	-0.016	-0.186	0.852
	(0.060)	(0.062)			
Risk loving	5.604	5.623	-0.019	-0.221	0.825
	(0.060)	(0.062)			
Altruism	4.814	4.916	-0.102	-1.017	0.309
	(0.067)	(0.075)			
Trust	5.096	5.070	0.026	0.234	0.815
	(0.075)	(0.080)			
PGS of Cognitive Ability	-0.009	-0.011	0.002	0.302	0.763
	(0.005)	(0.005)			
PGS of Depression	32.591	32.671	-0.080	0.466	0.641
	(0.131)	(0.112)			
PGS of Delay Discounting	0.074	0.074	0.000	-0.010	0.992
	(0.002)	(0.003)			
PGS of Reproduction Preference	0.028	0.031	-0.004	-0.520	0.604
	(0.005)	(0.005)			

As a second strategy to address the potential population stratification problem, we include a total of 42 individual ancestral composition variables based on each respondent’s genetic data as additional control variables (The 42 ancestries are, from high to low: Northern Han, Southern Han, Mongolian, Naxi/Yi, Japanese, Gaoshan, Korean, Dai, She, Kinh, Tibetan, Tungus, Ashkenazi, Balkan, Bantusa, Bengali, Cambodian, Egyptian, English, Eskimo, Finnish/Russian, French, Hungarian, Iranian, Kyrgyz, Lahu, Mala, Mayan, Mbuti, Miao/Yao, Papuan, Pima, Sardinian, Saudi, Sindhi, Somali, Spanish, Thai, Uygur, Uzbek, Yakut, and Yoruba.). In our data, individual ancestry composition is estimated by using the ADMIXTURE program, developed by the Department of Human Genetics, University of California Los Angeles [64–65]. Table 3 reports the descriptive information of the top ten ancestries estimated in our sample. In all subsequent analyses, we include a full set of 42 ancestral controls unless otherwise noted.

**Table 3:** Descriptive statistics of top 10 ancestries.

Ancestry/Population	Mean	Std.Dev.	Min	Max
Northern Han	0.5530	0.2963	0.0000	0.9996
Southern Han	0.2579	0.2645	0.0000	0.9996
Mongolian	0.0604	0.1124	0.0000	0.7774
Naxi/Yi	0.0292	0.0610	0.0000	0.9996
Japanese	0.0202	0.0369	0.0000	0.2173
Gaoshan	0.0084	0.0171	0.0000	0.1163
Korean	0.0075	0.0105	0.0000	0.0575
Dai	0.0070	0.0203	0.0000	0.1696
She	0.0056	0.0096	0.0000	0.0540
Kinh	0.0054	0.0147	0.0000	0.1260

Source: author’s calculation.

#### 4.3.3. Exclusion

The exclusion criterion requires that the genetic IV of PGS_Height have no direct effect on income through pleiotropy. In our design, if some of the SNPs used to construct PGS_Height can directly affect the outcome variable of interest (i.e., income), then the exclusion condition of the genetic IV would be violated [23]. Although it is empirically impossible to prove the null hypothesis that PGS_Height is uncorrelated with the error term in 2SLS regressions by using a single instrumental variable, we perform a series of tests assessing the validity of the exclusion restriction assumption [62–63].

First, we follow VanderWeele and colleagues [62] and estimate a reduced-form model, that of log earnings on the genetic score of height (*PGS_Height*), while excluding the endogenous explanatory variable, *Height*, from the model. If height does not have a causal effect on (log) earnings, then one would expect the genetic score of height to have little impact on (log) earnings. The results, reported in Table 4 verify this expectation. The estimated coefficients on *PGS_Height* in all three specifications are small and statistically insignificant, suggesting little predictive power of the genetic IV for the outcome variable.

**Table 4:** Additional tests of the exclusion restriction assumption.

	(1)	(2)	(3)
	ln(income)	ln(income)	ln(income)
*PGS_Height*	0.0047	0.0057	0.0046
	(0.0436)	(0.0403)	(0.0435)
Male		0.027	0.0277
		(0.0364)	(0.0392)
Age		0.2042***	0.2031***
		(0.0188)	(0.0202)
Age^2		-0.0023***	-0.0023***
		(0.0003)	(0.0003)
Years of Schooling		0.0498***	0.0525***
		(0.0090)	(0.0096)
Constant	11.5921***	6.8570***	6,236.7441**
	(0.0328)	(0.3264)	(2,836.0718)
Additional Controls of Personality and Other Polygenic Scores	Yes	Yes	Yes
Province FE	No	No	Yes
Ancestral Controls	No	No	Yes
Observations	3,427	3,427	3,427
R-squared	0.0006	0.1468	0.1845

***

**, and

* indicate statistical significance at the 1%, 5%, and 10% levels, respectively.

Second, we compare the magnitude of the coefficients of *Height* in the OLS and the IV models. If our genetic IV satisfies the exclusion restriction, one would expect to see ${\hat{\beta }}_{IV}<{\hat{\beta }}_{OLS}$ under the assumption that there is positive unmeasured confounding of height and income [63]. Our results reported in Table 5 (column 2, ${\hat{\beta }}_{OLS}$ *=* 0.0103) and Table 7 (column 1, ${\hat{\beta }}_{IV}$ = 0.0056) are consistent with this expectation (i.e., 0.0056 < 0.0103). This result indicates no obvious violation of the exclusion restriction assumption.

**Table 5:** OLS results.

Variables	(1)	(2)	(3)	(4)	(5)
	Pooled	Pooled	Male	Female	Age 30–50
	ln(income)	ln(income)	ln(income)	ln(income)	ln(income)
Height	0.0130***	0.0103***	0.0138***	0.0084**	0.0128***
	(0.0021)	(0.0023)	(0.0030)	(0.0037)	(0.0035)
Male	-0.0105	0.0167	-	-	0.0036
	(0.0355)	(0.0382)	-	-	(0.0566)
Age	0.1994***	0.1995***	0.2347***	0.1732***	0.1888**
	(0.0181)	(0.0194)	(0.0269)	(0.0290)	(0.0767)
Age^2	-0.0023***	-0.0023***	-0.0028***	-0.0018***	-0.0021**
	(0.0003)	(0.0003)	(0.0004)	(0.0004)	(0.0010)
Years of schooling	0.0525***	0.0555***	0.0249**	0.0880***	0.0522***
	(0.0088)	(0.0093)	(0.0124)	(0.0148)	(0.0134)
*Personality traits*:					
Risk Loving	-	0.0542***	0.0415***	0.0734***	0.0374***
	-	(0.0092)	(0.0122)	(0.0147)	(0.0139)
Altruism	-	0.0011	0.0033	0.0025	0.0098
	-	(0.0081)	(0.0111)	(0.0124)	(0.0120)
Trust	-	0.0006	-0.0094	0.0090	-0.0001
	-	(0.0073)	(0.0097)	(0.0116)	(0.0113)
*Additional genetic factors*:					
Cognitive ability	-	-0.1138	-0.1152	-0.0079	-0.0325
	-	(0.1128)	(0.1519)	(0.1751)	(0.1699)
Depression	-	-0.0050	-0.0071	0.0002	-0.0144**
	-	(0.0043)	(0.0057)	(0.0068)	(0.0066)
Delay discounting	-	-0.5264**	-0.6943**	-0.1819	-0.3947
	-	(0.2218)	(0.2980)	(0.3415)	(0.3257)
Reproduction preference	-	0.0092	-0.0318	-0.0686	0.0583
	-	(0.1144)	(0.1562)	(0.1745)	(0.1678)
Constant	4.3258***	4,431.2922	3,905.0754	4,307.0775	6,813.2636*
	(0.4841)	(2,756.9803)	(3,648.9270)	(4,355.0797)	(4,118.1286)
Province FE	Yes	Yes	Yes	Yes	Yes
Ancestral controls	Yes	Yes	Yes	Yes	Yes
Observations	3,427	3,427	1,922	1,505	1,843
R-squared	0.1631	0.2187	0.2344	0.2829	0.1835

***

**, and

* indicate statistical significance at the 1%, 5%, and 10% levels, respectively. In all models, we control for province fixed effects and 42 individual ancestry composition variables.

It is worth noting that Böckerman et al. and Palmer et al. point out that the use of polygenic scores can significantly reduce the risk of pleiotropy compared with a single gene variant or SNP [25,66]. Böckerman et al. also explicitly demonstrate the robustness of the causal effect of height on earnings after adjusting for potential pleiotropy [25], which lends further support to the validity of using the polygenic score of height as an IV for the observed height in an MR setting.

### 4.4. Personality traits and other genetic factors

In **X**, we include personality traits (i.e., risk attitude, altruism and trust) and additional genetic controls (i.e., cognitive ability, depression, delay discounting, and reproduction preference) for two reasons. First, personality characteristics (also known as noncognitive or soft skills) have been found to play crucial roles in determining one’s labor market success [25,67–68]. Second, it is well-known that cognitive ability is closely related to labor market success [16]. Although the phenotypic variable of cognitive ability is not available in the current study, the inclusion of its proxy—the polygenic scores of cognitive ability, can help mitigate the potential omitted variable bias [48]. Similarly, if any of the behavioral traits of depression, delay discounting, or reproduction preference contributes to one’s labor market success, then although their phenotypic variables are not available, it is intuitive to also include their genetic proxies (i.e., polygenic scores of depression, delay discounting, and reproduction preference) to avoid the omitted variable bias, and further strength our empirical results.

## 5. Results

Turing to our empirical results, this section first documents the existence of a height premium using OLS regressions, with various specifications that have been adopted in previous studies. By sequentially adding more explanatory variables in the model, these OLS estimates help shed light on what is being captured in the estimated height premium. We then use the genetic IV to estimate the causal effect of height, to see how genetic height affects one’s earnings while netting out the influence of confounding factors.

### 5.1. OLS results: Is there a height premium?

Table 5 reports main results of estimating Eq (1) by OLS. Overall, these results are consistent with those of previous observational studies conducted in China [20–21], which suggest a statistically significant and positive association between one’s adult height and wage income.

Model 1 and 2 presents results for the full sample. As a benchmark, Model 1 controls for gender, age, years of schooling, province fixed effects, and individual ancestral composition. It estimates that one additional cm in height is associated with a 1.30% increase in annual salary income, which is similar to the findings of Gao and Smyth [20] and Yamamura et al. [21] using Chinese data.

Model 2 further includes a set of personality traits (namely, risk-loving, altruism and trust) and additional genetic markers (including polygenic scores for cognitive ability, depression, delay discounting, and reproduction preference) that can potentially affect one’s labor-market performance in the regression. The estimated height premium declines–a 1-cm increase in height is associated with a 1.03% increase in annual income. It is worth noting that higher risk-loving preference and lower polygenic score of delay discounting (i.e., genetically less hyperbolic or temporal discounting, and preferring future return or gratification rather than immediate rewards) are positively associated with annual income. Results in Table 6, panel A also suggest that height is significantly correlated with risk-loving and altruism (columns 3–6), even after controlling for the genetic determinant of height (i.e., *PGS_Height*). Table 6, panel B further explores the co-occurrence of height and those polygenic scores, and interestingly it appears that higher genetic cognitive ability (column 2) and lower genetic risk of depression (column 4) are associated with higher genetic height (but not higher observed height), which is consistent with the simultaneous neural and height growth discussed by Tanner [41] and Thompson and Potter [42]. These results imply that one should control for those polygenic scores in the regression to avoid and/or mitigate the omitted-variable bias and potential pleiotropy whenever possible. In the following models, we include these personality traits and additional genetic markers unless otherwise noted.

**Table 6:** Relationship between height and additional control variables.

**A. Correlations between height and personality traits**								
	-1	-2	-3	-4	-5	-6	-7	-8
	Years of Schooling	Years of Schooling	Risk Loving	Risk Loving	Altruism	Altruism	Trust	Trust
Height	-0.0018	-0.0008	0.0294***	0.0298***	0.0247***	0.0241***	-0.0124	-0.0115
	-0.0062	-0.0065	-0.0065	-0.0067	-0.0076	-0.0079	-0.0083	-0.0086
*PGS_Height*	-	-0.1119	-	-0.0986	-	-0.0694	-	0.0508
	-	-0.1162	-	-0.1208	-	-0.1416	-	-0.1547
Constant	16.7378***	16.4893***	0.6234	0.5003	0.7132	0.7736	7.2942***	7.1546***
	-1.0582	-1.114	-1.1026	-1.157	-1.2967	-1.3559	-1.4131	-1.4808
Observations	3,427	3,427	3,427	3,427	3,427	3,427	3,427	3,427
R-squared	0.0001	0.0007	0.013	0.0128	0.0067	0.0062	0.0014	0.0012
**B. Correlations between height and other PGS**								
	-1	-2	-3	-4	-5	-6	-7	-8
Variables	PGS: Cognitive Ability	PGS: Cognitive Ability	PGS: Depression	PGS: Depression	PGS:	PGS:	PGS: Reproduction Preference	PGS: Reproduction Preference
					Delay Discounting	Delay Discounting		
Height	0.0003	0	-0.0164	-0.0183	-0.0002	-0.0002	-0.0004	-0.0005
	-0.0005	-0.0005	-0.0182	-0.0121	-0.0003	-0.0003	-0.0005	-0.0005
*PGS_Height*	-	0.0206**	-	-0.2373**	-	0.0028	-	0.0059
	-	-0.0096	-	-0.1118	-	-0.005	-	-0.0096
Constant	-0.0578	-0.0026	34.7815***	35.6082***	0.1066**	0.1135**	0.0965	0.1112
	-0.0863	-0.0915	-3.0966	-2.0798	-0.0462	-0.0478	-0.0887	-0.0918
Observations	3,427	3,427	3,427	3,427	3,427	3,427	3,427	3,427
R-squared	0.0002	0.0032	0.0005	0.0028	0.0003	0.0005	0.0004	0.0007

***

**, and

* indicate statistical significance at the 1%, 5%, and 10% levels, respectively.

Model 3 and 4 of Table 4 report OLS estimates of male and female respondents, respectively. Similar to the results of Gao and Smyth [20], the estimated return to height is more pronounced among males than among females. Model 5 analyzes the subsample of prime working-age individuals (age 30–50), in which a 1-cm increase in height is associated with a 1.28% increase in annual income, higher than that of the full sample.

Taken together, the OLS results reported in Table 5 indicate that height has a statistically and economically significant effect on (the log of) annual salary income, even after controlling for a large set of covariates that are likely to be correlated with height. Since it is difficult to control for all potential confounding factors, it remains unclear whether the estimated height premium will further decline when more covariates are included in the OLS model. Assessing this issue from another angle, the next subsection aims to estimate the economic return to genetic height, using genetic markers as an IV for observed height.

### 5.2. 2SLS results: Is the height premium causal?

Table 7 presents the main results of two-stage least squares (2SLS) regressions, with odd-numbered columns reporting results of second-stage regressions and even-numbered ones reporting first-stage regression results. In all models, the polygenic score of height (*PGS_Height*) is used as the sole instrument for observed height. An important finding emerging from Table 7 is that the 2SLS estimates of the impact of height become much smaller and are no longer statistically significant at any conventional level, implying little causal link between genetic height and earnings. This finding, in turn, suggests that labor-market discrimination is unlikely to be the key factor driving the observed height premium documented by OLS regressions in Table 5.

**Table 7:** 2SLS results.

Outcome variables	Model 1: Pooled		Model 2: Male		Model 3: Female		Model 4: Age 30–50	
	(1)	(2)	(3)	(4)	(5)	(6)	(7)	(8)
	2SLS	First Stage	2SLS	First Stage	2SLS	First Stage	2SLS	First Stage
	Ln(income)	Height	Ln(income)	Height	Ln(income)	Height	Ln(income)	Height
Height	0.0056	-	0.0063	-	0.0029	-	0.0075	-
	(0.0107)	-	(0.0172)	-	(0.0137)	-	(0.0152)	-
Male	0.0217	10.1720***	-	-	-	-	0.0031	10.5547***
	(0.0377)	(0.3530)	-	-	-	-	(0.0554)	(0.5591)
Age	0.2106***	-0.1478	0.2482***	-0.2206	0.1771***	-0.1977	0.2033***	0.0973
	(0.0195)	(0.1608)	(0.0264)	(0.2207)	(0.0290)	(0.2440)	(0.0765)	(0.7687)
Age^2	-0.0024***	0.0004	-0.0030***	0.0013	-0.0019***	0.0014	-0.0023**	-0.0022
	(0.0003)	(0.0024)	(0.0004)	(0.0032)	(0.0004)	(0.0036)	(0.0010)	(0.0101)
Years of schooling	0.0495***	0.0419	0.0212*	0.0464	0.0755***	-0.0428	0.0509***	0.0165
	(0.0092)	(0.0848)	(0.0121)	(0.1146)	(0.0142)	(0.1318)	(0.0132)	(0.1326)
Risk loving	0.0537***	0.4047***	0.0434***	0.3397***	0.0693***	0.5015***	0.0340**	0.4152***
	(0.0100)	(0.0833)	(0.0135)	(0.1128)	(0.0153)	(0.1290)	(0.0145)	(0.1331)
Altruism	0.0012	0.1350*	0.0036	0.0677	0.0017	0.1835	0.0093	0.1253
	(0.0080)	(0.0737)	(0.0107)	(0.1019)	(0.0123)	(0.1116)	(0.0115)	(0.1161)
Trust	0.0010	-0.2059***	-0.0100	-0.1447	0.0115	-0.2547**	0.0013	-0.0918
	(0.0076)	(0.0669)	(0.0096)	(0.0902)	(0.0120)	(0.1044)	(0.0109)	(0.1089)
Cognitive ability	-0.1092	-0.1979	-0.1027	-0.1497	-0.0250	-0.1688	-0.0316	-1.8792
	(0.1104)	(1.0313)	(0.1470)	(1.4084)	(0.1673)	(1.5711)	(0.1634)	(1.6424)
Depression	-0.0052	0.0323	-0.0070	0.0091	-0.0002	0.0410	-0.0145**	0.0043
	(0.0042)	(0.0407)	(0.0055)	(0.0547)	(0.0065)	(0.0631)	(0.0063)	(0.0647)
Delay discounting	-0.5255**	-2.3509	-0.7054**	-0.8149	-0.1790	-3.1801	-0.3644	-1.8665
	(0.2182)	(2.0091)	(0.2869)	(2.7791)	(0.3279)	(2.9905)	(0.3129)	(3.1492)
Reproduction preference	0.0148	-1.3113	-0.0257	0.8948	-0.0201	-4.3051***	0.0949	-2.2624
	(0.1125)	(1.0587)	(0.1519)	(1.4567)	(0.1768)	(1.5927)	(0.1636)	(1.6250)
Constant	109.9583	96.1696	12.0476	86.0966	-0.4013	39.8194	3.8526	15.5517
	(80.6906)	(200.3519)	(146.3101)	(204.1028)	(12.8593)	(210.7858)	(40.5838)	(174.1510)
Province FE	Yes	Yes	Yes	Yes	Yes	Yes	Yes	Yes
Ancestral controls	Yes	Yes	Yes	Yes	Yes	Yes	Yes	Yes
*Instrumental Variable*:								
*PGS_Height*	-	3.9284***	-	3.2287***	-	4.6578***	-	3.6183***
	-	(0.3947)	-	(0.5302)	-	(0.6137)	-	(0.6200)
First-stage F statistic	85.1053		29.9235		52.1446		34.3092	
p-value	0.0000		0.0000		0.0000		0.0000	
Observations	3,427		1,922		1,505		1,843	

***

**, and

* indicate statistical significance at the 1%, 5%, and 10% levels, respectively. In all models, we control for province fixed effects and 42 individual ancestry composition variables.

More specifically, the first model of full sample (column 1) suggests that the impact of height on annual income identified by Mendelian randomization of height-related genes upon conception is very small compared to its OLS counterpart (Table 5, column 2)–other things being equal, an additional cm in (genetic) height increases one’s annual salary by less than 0.6%. Such an impact is not statistically significant at any conventional level, echoing the finding of Böckerman et al. using Finnish data [25]. The corresponding first-stage regression (column 2) reveals no sign of the weak-IV problem: the IV used, *PGS_Height*, has a very strong predictive power for height, with an associated F-statistic of 85.1053, far exceeding the rule-of-thumb value of 10 [69]. Models 2–4 further perform 2SLS estimations by subsamples of males, females, and prime working-age participants, respectively, but neither model yields a statistically or economically significant height premium.

As additional checks, we perform 2SLS by including the quadratic form of *PGS_Height* (i.e., *PGS_Height^2*) as an additional genetic IV to the above models again. The key results remain robust (reported in S1 Appendix in Table A1), whereas first-stage regression results show little predictive power of *PGS_Height^2* (i.e., insignificant in all models), implying a linear effect of *PGS_Height* on observed height. It is worth pointing out that the use of two genetic IVs facilitates an overidentification test for the validity of the exclusion restriction. For example, in Model 1 of the full sample (S1 Appendix in Table A1, column 1–2), results of the standard overidentification test (Sargan statistic = 2.2653, *p* = 0.1323) reveal no sign of violation of the exclusion restriction, lending further support to the IV validity of *PGS_Height*.

## 6. Concluding remarks

The income disparities associated with one’s physical appearance (such as height, stature, BMI, facial appearance) have motivated a large body of studies that seek to identify the underlying causal mechanisms [33]. This paper picks up this agenda and uses very recent data from China to show that without correcting for endogeneity, body height is indeed associated with higher salary income. However, once we have instrumented the observed height with its genetic determinants (i.e., the polygenic score of height), such height premium vanishes. Taking advantage of the unique prosperous individual genotyping data available in our sample, we then explore and illustrate that genetic cognitive ability, genetic risk of depression, various personality traits, as well as remaining confounders that are correlated with height are likely to be those truly captured in the observed height premium.

While we find an economically insubstantial premium of height *per se*, which suggests that discrimination from the workplace may not be a crucial component to wage disparity with respect to height, we interpret these results as empirical support of channels through which both cognitive and non-cognitive skills are correlated with height during individual development. In other words, the observed height may act as a signal of beneficial circumstances for developing higher cognitive/non-cognitive skills during childhood or early-life [25]. This naturally suggests a role for redistributive policies to support children and youths from lower SES households. If individuals from households with limited resources do not reach their height and/or human capital potentials due to insufficient human capital investment in childhood, then simply implementing anti-discrimination laws in the labor market may not substantially reduce income inequality.

Before closing, there are several limitations of the present study. First, the current study is based on a sample of 3,427 observations, which may lack statistical power due to small sample size [70]. Second, as mentioned earlier, the WeGene sample we use in this study is not nationally representative with respect to the total population in China. More studies may be needed to confirm the generality of our findings. Third, the polygenic score of height in this paper is constructed as an aggregate measure of 697 SNPs, in which most of these SNPs are involved in skeletal growth and bone development from pathway analyses [47]. Although this strongly suggests that the LATE presented in the current study can be mainly interpreted as resulting from the main biological pathways of skeletal growth/bone development, we lack a comprehensive understanding of the potential different LATEs through other minor biological mechanisms due to data limitations, and it can be an intriguing direction for future research. Fourth, although we find that height and cognitive/non-cognitive abilities are correlated at the genetic level, the underlying biological and/or environmental mechanisms are still unclear. One possible explanation of that may be *positive selection* from the realm of evolutionary biology, in which beneficial traits (i.e. those that can make carriers survive and reproduce more successfully) tend to be selected by exogenous environments and become more frequent in populations over time [71]. Future research may look for supporting evidence or explore additional explanation to gain a deeper understanding of the co-occurrence of height and cognitive/non-cognitive abilities in modern humans.

## Supporting information

S1 Appendix (DOCX)
