# Supplementary material for: What is creating the height premium? New evidence from a Mendelian randomization analysis in China
Source: PLoS One. 2020 Apr 10;15(4):e0230555. doi: 10.1371/journal.pone.0230555 (PMC7147798; doi:10.1371/journal.pone.0230555)
Supplement: S1 Appendix — (DOCX) [file pone.0230555.s001.docx]

# Appendix:

**Table A1. Additional 2SLS Results**

| Outcome variables | Model 1: Pooled | | Model 2: Male | | Model 3: Female | | Model 4: Age 30-50 | |
| --- | --- | --- | --- | --- | --- | --- | --- | --- |
|  | (1) | (2) | (3) | (4) | (5) | (6) | (7) | (8) |
|  | 2SLS | First Stage | 2SLS | First Stage | 2SLS | First Stage | 2SLS | First Stage |
|  | Ln(income) | Height | Ln(income) | Height | Ln(income) | Height | Ln(income) | Height |
| Height | 0.0053 | - | 0.0060 | - | 0.0028 | - | 0.0081 | - |
|  | (0.0107) | - | (0.0165) | - | (0.0137) | - | (0.0152) | - |
| Male | 0.0219 | 10.1303*** | - | - | - | - | 0.0030 | 10.7335*** |
|  | (0.0377) | (0.3530) | - | - | - | - | (0.0554) | (0.5608) |
| Age | 0.2107*** | -0.1498 | 0.2490*** | -0.2123 | 0.1770*** | -0.1942 | 0.2034*** | 0.1052 |
|  | (0.0195) | (0.1608) | (0.0264) | (0.2206) | (0.0290) | (0.2444) | (0.0765) | (0.7693) |
| Age^2 | -0.0024*** | 0.0005 | -0.0030*** | 0.0012 | -0.0019*** | 0.0013 | -0.0023** | -0.0023 |
|  | (0.0003) | (0.0024) | (0.0004) | (0.0032) | (0.0004) | (0.0036) | (0.0010) | (0.0101) |
| Years of schooling | 0.0494*** | 0.0428 | 0.0211* | 0.0483 | 0.0755*** | -0.0434 | 0.0509*** | 0.0170 |
|  | (0.0092) | (0.0848) | (0.0121) | (0.1145) | (0.0142) | (0.1319) | (0.0132) | (0.1326) |
| Risk loving | 0.0531*** | 0.4033*** | 0.0404*** | 0.3385*** | 0.0694*** | 0.5028*** | 0.0338** | 0.4160*** |
|  | (0.0099) | (0.0833) | (0.0133) | (0.1127) | (0.0153) | (0.1291) | (0.0145) | (0.1332) |
| Altruism | 0.0010 | 0.1347* | 0.0040 | 0.0671 | 0.0017 | 0.1839 | 0.0092 | 0.1238 |
|  | (0.0080) | (0.0737) | (0.0107) | (0.1018) | (0.0123) | (0.1117) | (0.0116) | (0.1162) |
| Trust | 0.0015 | -0.2047*** | -0.0090 | -0.1424 | 0.0115 | -0.2556** | 0.0014 | -0.0890 |
|  | (0.0076) | (0.0669) | (0.0096) | (0.0901) | (0.0120) | (0.1045) | (0.0109) | (0.1092) |
| Cognitive ability | -0.1108 | -0.1954 | -0.1104 | -0.1409 | -0.0250 | -0.1664 | -0.116 | -1.9053 |
|  | (0.1105) | (1.0313) | (0.1469) | (1.4075) | (0.1673) | (1.5719) | (0.1634) | (1.6442) |
| Depression | -0.0052 | 0.0338 | -0.0071 | 0.0097 | -0.0002 | 0.0389 | -0.0145** | 0.0060 |
|  | (0.0042) | (0.0407) | (0.0055) | (0.0547) | (0.0065) | (0.0634) | (0.0063) | (0.0648) |
| Delay discounting | -0.5176** | -2.2808 | -0.7002** | -0.7252 | -0.1796 | -3.2384 | -0.3634 | -1.8917 |
|  | (0.2182) | (2.0105) | (0.2867) | (2.7777) | (0.3278) | (2.9973) | (0.3131) | (3.1513) |
| Reproduction preference | 0.0179 | -1.3392 | -0.0356 | 0.8695 | -0.0206 | -4.2768*** | 0.0963 | -2.3197 |
|  | (0.1125) | (1.0592) | (0.1517) | (1.4557) | (0.1768) | (1.5958) | (0.1637) | (1.6308) |
| Constant | 30.3317 | 93.9739 | 14.3106 | 81.1474 | -0.3156 | 90.0193 | 3.6010 | 66.3959 |
|  | (184.1224) | (200.3681) | (183.8341) | (203.9828) | (12.8384) | (95.0355) | (40.2068) | (110.4366) |
| Province FE | Yes | Yes | Yes | Yes | Yes | Yes | Yes | Yes |
| Ancestral controls | Yes | Yes | Yes | Yes | Yes | Yes | Yes | Yes |
| *Instrumental Variables:* | |  |  |  |  |  |  |  |
| PGS_Height | - | 4.6452*** | - | 4.7589*** | - | 4.2329*** | - | 4.1618*** |
|  | - | (0.8410) | - | (1.0850) | - | (1.4314) | - | (1.3526) |
| PGS_Height^2 | - | 0.5891 | - | 1.2949 | - | -0.3301 | - | 0.4393 |
|  | - | (0.6103) | - | (0.8012) | - | (1.0047) | - | (0.9717) |
| First-stage F statistic | 46.0529 | | 27.1931 | | 43.0970 | | 41.9801 | |
| p-value | 0.0000 | | 0.0000 | | 0.0000 | | 0.0000 | |
| Sargan Statistic | 2.2653 | | 2.6118 | | 0.1486 | | 0.4718 | |
| p-value | 0.1323 | | 0.1061 | | 0.6999 | | 0.4922 | |
| Observations | 3,427 | | 1,922 | | 1,505 | | 1,843 | |

Notes: ***, **, and * indicate statistical significance at the 1%, 5%, and 10% levels, respectively. In all models, we control for province fixed effects and 42 individual ancestry composition variables.
